# Identification of *RppSLN* from an Elite Landrace: A Major Locus Conferring Resistance to Southern Corn Rust in Maize (*Zea mays* L.)

**DOI:** 10.3390/plants13223227

**Published:** 2024-11-16

**Authors:** Yufei Wang, Shuai Ma, Dengfeng Zhang, Chunhui Li, Lin Chen, Bin Tang, Yixin An, Xuyang Liu, Guanhua He, Yunsu Shi, Yu Li, Tianyu Wang, Deguang Yang, Yongxiang Li

**Affiliations:** 1 College of Agriculture, Northeast Agricultural University, Harbin 150006, China; 2 State Key Laboratory of Crop Gene Resources and Breeding, Institute of Crop Sciences, Chinese Academy of Agricultural Sciences, Beijing 100081, Chinalichunhui@caas.cn (C.L.); tangbin@hunaas.cn (B.T.); an1196917412@163.com (Y.A.);; 3 Institute of Grassland, Flowers and Ecology, Beijing Academy of Agriculture and Forestry Sciences, Beijing 100097, China

**Keywords:** maize (*Zea mays* L.), southern corn rust, landrace, *RppSLN*

## Abstract

Southern corn rust (SCR) is one of the most destructive foliar diseases in maize (*Zea mays* L.), resulting in significant yield losses. Therefore, the continuous identification of disease-resistant germplasm and the deployment of resistant hybrids is essential for durably controlling SCR. The objective of this research was to identify and characterize resistance loci against SCR in maize to expand disease management strategies. Here, we identified a maize landrace with high resistance to SCR ‘Silunuo’ (SLN) approaching complete immunity. We backcrossed it with a susceptible inbred line, N531, to generate a stable SCR-resistant introgression line N531_R. By crossing it with F35 (a susceptible inbred line), we created a large F_2_ segregating population and mapped a major SCR-resistant locus on chromosome 10, known as *RppSLN*. Based on the genome assembly and annotation, we found that *RppSLN* harbors two NBS-LRR (nucleotide binding site–leucine-rich repeat) genes, namely *Zmays10G000430* and *Zmays10G000440*. These NBS-LRR genes were significantly induced during artificial inoculation with *Puccinia polysora*, suggesting that they might be candidate genes collectively contributing to the resistance level at this locus. In conclusion, this study identified a major SCR resistance locus directly isolated from a landrace, providing valuable support and information for expanding new disease-resistant germplasms and promoting the utilization of landraces.

## 1. Introduction

Southern corn rust (SCR) is a highly destructive airborne disease caused by *Puccinia polysora* (*P. polysora*), leading to significant maize (*Zea mays* L.) yield losses. SCR has spread to most major corn-producing regions worldwide, including Asia, the Americas, and Africa [1]. The disease has caused significant yield losses during outbreaks, with losses often reaching 50% [2]. In recent years, global climate change has created conditions favorable for disease development, exacerbating SCR and causing its gradual spread to higher-latitude regions [3]. In China, SCR affected 5.289 million hectares, resulting in a loss of 756 million kilograms of corn in 2015 alone [4]. The scarcity of resistance genes and restricted genetic diversity are the main reasons for SCR epidemics [5]. Therefore, identifying diverse-resistant germplasm resources and additional resistance loci is essential for sustainable disease control [6,7,8].

Plants have developed two forms of resistance mechanisms, which are complete resistance, governed by a single gene (qualitative resistance) [9], and incomplete resistance, governed by multiple genes with partial effects (quantitative resistance) [10]. Resistance genes (R-genes) confer race-specific qualitative resistance against biotrophic pathogens, such as *P. polysora*, which can survive within host plant cells for several days [11,12,13]. However, resistance from a single R-gene is often short-lived. Similarly to other plant pathogenic microbes, *P. polysora* has a rapid mutation rate, allowing it to overcome maize resistance. For example, the *Rpp9* gene once provided effective resistance to SCR in the southern United States, but it has since been overcome by a new race of *P. polysora* [14]. This situation underscores the urgent need to discover new resistant genes to combat the emergence of new physiological races and their variations.

Currently, nearly all materials used to identify resistance to SCR for breeding originated from inbred lines, some of which belong to the P group [15], such as K22, Q319, and X178, carrying resistance sources traced to the Pioneer hybrid PH78599 [16,17]. The *RppC* resistance gene, originating from the tropical material CML496, is present in 53.3% of the top 30 commercial hybrids in China, encompassing 72.5% of the total planted area [18]. However, to prevent resistance breakdown caused by limited resistance germplasm and protect maize yields, it is essential to investigate new germplasm resources and resistance loci.

Landraces are the foundation for the development of modern inbred lines, with their rich genetic diversity providing a vast gene pool for crop breeding and improvement. Here, we discovered a unique landrace exhibiting high resistance to SCR, ‘Silunuo’ (SLN), with many primordial traits having restricted distribution in southwest Yunnan in China. Therefore, we developed a stably resistant SCR introgression line named N531_R by using the resistant landrace SLN as a donor and the susceptible inbred line N531 as a recurrent parent, following multiple generations of backcrossing and selfing. Subsequently, we identified a single QTL associated with SCR, *RppSLN*, narrowing it down to a 38 kb physical interval on chromosome 10, employing an F_2_ population by crossing the resistant line N531_R with the susceptible line F35. Within this interval, we identified two candidate genes that exhibited similar expression patterns when subjected to artificial inoculation with SCR. This study directly identified SCR resistance genes from a landrace variety and further investigation into the mechanisms underlying these candidate genes will be conducted in future research. The aim of this study is to identify resistance loci from landraces and, in conjunction with transcriptome analysis, to recognize candidate genes, thereby providing theoretical support for resistance to SCR.

## 2. Materials and Methods

### 2.1. Plant Materials

‘Silunuo’ (SLN), collected from the Menghai region of Yunnan in China, is a landrace with several primordial traits, including a well-developed root system and strong adaptability [19]. Due to its high resistance to SCR observed in our field phenotyping research, SLN is an elite genetic resource for SCR resistance. We crossed a susceptible inbred line Nong531 (N531) with SLN and conducted six rounds of backcrossing to generate BC_6_. Finally, we obtained introgression lines highly resistant and susceptible to SCR, a pair of NILs (near-isogenic lines), designated as N531_R and N531_S, respectively. We crossed a susceptible inbred line F35 with N531_R to generate F_1_. We then self-pollinated to produce an F_2_ population consisting of 183 individual plants for preliminary mapping. Furthermore, we expanded our efforts by constructing a large-scale F_2_ segregating population containing 2253 individual plants.

### 2.2. Phenotyping and Field Trials

Disease scores were recorded under spontaneous disease conditions using the Stakman infection-type scale with a 1–9 rating [5,20,21]. Disease severity assessments were conducted three times at weekly intervals, starting two weeks after pollination. The disease level was evaluated using a five-point rating scale, with N531_R serving as the resistance control and N531_S as the susceptible control. The disease phenotypic was classified into 5 levels, with Level 1—highly resistant (HR), no disease lesions or only a hypersensitivity response; Level 3—resistant (R), no more than 25% of the leaf area infected; Level 5—medium resistance (MR), leaf lesions infecting between 26% and 50% of the leaf area; Level 7—susceptible (S), leaf lesions infecting between 51% and 75% of the leaf area; and Level 9—highly susceptible (HS), leaf lesions infecting more than 70% of the leaf area, potentially leading to plant death.

All maize plants were planted in Yacheng, Sanya City, Hainan Province, China (2020 HN, 18.36° N, 109.17° E). Field management followed local maize production practices. The F_2_ QTL-mapping population (N531_R×F35) used for genetic segregation was sown uniformly in 3 m long rows, with 15 individuals per row, and the rows were spaced 60 cm apart.

### 2.3. Genotyping

Genomic DNA was extracted from young leaf tissues using the CTAB method [22]. The F_2_ population for rough mapping and their parents were genotyped using the GenoBaits Maize 1K Panel (MolBreeding Biotechnology Company Co., Ltd., Shijiazhuang, China), which uniformly covered the genome and was aligned to the B73_v3 reference genome map. A total of 1354 SNP markers were identified on the basis of genotyping by target sequencing (GBTS) technology [23]. Additional filtering based on MAF, missing data (NA), and heterozygosity (Het) values was conducted using Plink v.1.9.0 [24] and VCFtools v0.1.15 [25]. Low minor allelic gene frequency values (MAF < 0.05), high missing data values (NA > 0.1), and high heterozygosity values (Het > 0.8) were filtered out from the original sequencing data, along with variants with monomorphic markers between parents. After quality control, 532 polymorphic SNPs between the two parental lines were retained for analysis.

### 2.4. Linkage Map Construction and QTL Mapping

QTL IciMapping v4.2.53 was used to build linkage maps and perform QTL mapping [26]. Individuals from the F_2_ population with filtered polymorphic SNP markers were used to build linkage maps, and inclusive composite interval mapping (ICIM) methods were utilized for QTL mapping. A logarithm of odds (LOD) threshold of 2.5 was adopted for considering a significant QTL. Stepwise regression was used to detect the percentages of phenotypic variance explained (PVE) by individual QTL and additive effects at the LOD peaks.

### 2.5. Genome Assembly and Annotation

High-quality DNA samples were extracted from two inbred lines, N531_R and its recurrent parent, N531, in 2-week-old seedlings to perform whole-genome sequencing. The de novo genome assembly process involved the three following steps: primary assembly, Hi-C scaffolding, and polishing. Genome assembly was performed through a sequencing library constructed for PacBio sequencing, Illumina sequencing, and BioNano optical mapping. For the generation of Hi-C data, genomic DNA was sequenced on an Illumina HiSeq 4000 platform and subsequently purified to obtain qualified DNA products suitable for library construction. Repeat regions in the N531_R and N531 genomes were identified through de novo predictions and homology-based annotation using RepeatModeler v2.0.1 [27]. Gene annotation combined ab initio training with evidence-based predictions for homolog evidence-based predictions, along with RepeatMasker v4.1.1 [28] and HelitronScanner v1.0 [29] for identifying repeat regions. For homolog evidence-based predictions, protein sequences from B73, Mo17, *Arabidopsis thaliana*, *Oryza sativa*, *Setaria italica*, and *Sorghum bicolor* were aligned with the N531_R and N531 genomes. For de novo predictions, we used Augustus v3.2.3 and Fgenesh v8.0.0a for training on the repeat masked N531_R and N531 genomes. For function annotation of the protein-coding genes, protein domains were identified using PfamScan v34.0 [30], and Gene Ontology (GO) term annotation was calculated by Blast2GO v6.0 [31] with default parameters.

### 2.6. Artificial Inoculation Traits

The two NILs of SCR-resistant N531_R and N531_S plants were grown in square boxes (5 cm × 5 cm) in a growth chamber (14 h of light/10 h of darkness). We evaluated the seedling growth status and chose healthy plants for the following artificial inoculation experiment. The inoculation of *P. polysora* spores was carried out using the leaf method [32]. The spores were brushed off susceptible leaves and transferred into sterilized ddH_2_O, then completely suspended in a 0.01% Tween 20 solution to achieve a concentration of approximately 5 × 10^5^ spores/mL. The spore suspension was evenly painted on all leaf surfaces at the 6-leaf stage. The inoculated seedlings were then placed in a dark greenhouse at 26 °C with 90% humidity for 12 h and then moved back to the growth chamber under normal growth conditions. The temperature ranged from 26 to 28 °C.

### 2.7. Real-Time Quantitative Analysis

Real-time quantitative analysis samples were selected from artificial inoculation experiments. We collected N531_R and N531_S samples at different time points, namely at 0 h (before inoculation) and at 12, 24, 48, and 96 h post-inoculation. To analyze the expression levels of candidate genes in N531_R and N531_S, total RNA was extracted from leaves using an RNAprep Pure Plant kit (Tiangen Biotech, Beijing, China). First-strand cDNA was synthesized using the TransGen^®^ II One Step gDNA Removal and cDNA Synthesis SuperMix kit (Transgen Biotech, Beijing, China). The maize actin gene (*Zm00001d012277*) was used as an internal reference. Relative changes in the expression of the three genes were analyzed using the 2^−∆∆CT^ method [33]. Each expression analysis included at least three biological replicates.

### 2.8. Transcriptome Analysis

RNA was extracted from leaves at 0, 12, 24, 48, and 96 h post-inoculation using Trizol reagent and quantified using a Nanodrop spectrophotometer (Thermo Fisher Scientific Inc., Wilmington, DE, USA) and a 2100 Bioanalyzer (Agilent Technologies, Santa Clara, CA, USA). Three biological replicates with sufficient RNA quality were used to construct RNA-seq libraries on the Illumina HiSeq platform (Illumina HiSeq X Ten) (Illumina, San Diego, CA, USA). Clean reads were mapped to the maize N531_R and N531 reference genomes using HISAT2 [34]. The alignment results were processed using StringTie v2.2.1 [35] software to assemble transcript isoforms and quantify expression values. Expression levels were calculated as fragments per kilobase of the exon model per million mapped reads (FPKMs) for both known and novel genes. Differential gene expression analysis was performed using the R package v4.4.0 DESeq2 to identify genes with a fold change (FC) ≥ 2 and a false discovery rate (FDR) ≤ 0.05. Hierarchical cluster analysis was performed on the differentially expressed genes (DEGs) to identify patterns of gene expression across different samples using Euclidean distance and complete linkage as the clustering method. Gene co-expression analysis was conducted to identify modules of co-expressed genes. Pairwise correlations between gene expression levels across samples were computed using Pearson’s correlation. A weighted co-expression network was then constructed using the WGCNA package in R, allowing for the identification of gene modules with highly correlated expression patterns.

### 2.9. Physiological and Biochemical Characteristics

We employed the same inoculation and sampling methods used for the transcriptomics analysis samples. Physiological and biochemical indicators assessed in this study included malondialdehyde (MDA) levels (nmol/mg prot), hydrogen peroxide (H_2_O_2_) content (µmol/mg prot), water-soluble sugar (WSS) (mg/mg), and proline content (mg/mg). Additionally, catalase (CAT) activity (U/mg) and peroxidase (POD) activity (U/mg) were measured. Testing was conducted using commercial assay kits from Beijing Solarbio Science & Technology Co., Ltd. (Beijing, China), following the manufacturer’s instructions [36].

### 2.10. Evaluation of Yield Breeding Value of RppSLN

The two NILs of N531_R and N531_S with SCR different resistance performances were used to evaluate the breeding value of the resistance locus of *RppSLN*. The resistant line N531_R, carrying the SCR-resistant allele of *RppSLN*, and the susceptible line N531_S, with the susceptibility locus, were crossed with the following four highly SCR-susceptible inbred lines: Chang7-2 (C7-2), Zheng58 (Z58), F35, and M35. This generated eight F_1_ hybrids (C7-2×N531_R, C7-2×N531_S, Z58×N531_R, Z58×N531_S, F35×N531_R, F35×N531_S, M35×N531_R, and M35×N531_S). To evaluate the yield performance of the hybrids in the field, all F_1_ hybrids were planted in Yacheng, Sanya City, Hainan Province, China (2020 HN, 18.36° N, 109.17° E), where the SCR is highly incident. The cultivation conditions for the F_1_ hybrids used for the yield assessment were the same as those for the F_2_ (N531_R×F35) population. Yield components were measured as follows: the ear weight was determined based on the total weight of a single ear, the kernel weight per ear was calculated as the weight of all corn grains in a single ear, and the hundred-kernel weight was measured by randomly selecting 100 uniformly sized grains. Each component was taken for three biological replicates, and all test data were imported into SPSS for statistical analysis. Statistical analysis was conducted using SPSS software v27. Data normality and homogeneity of variances were assessed prior to analysis. Significant differences between treatments were determined using one-way ANOVA (analysis of variance), followed by Tukey’s HSD (honestly significant difference) post hoc test for multiple comparisons. For data that did not meet the assumptions of ANOVA, the Kruskal–Wallis test was applied, followed by Dunn’s post hoc test for multiple comparisons. A *p*-value of less than 0.05 was considered statistically significant. Additionally, a chi-square test was used to analyze categorical data and to test for independence between variables.

## 3. Results

### 3.1. Identification of SCR-Resistant Locus RppSLN Isolated from Landrace

**Figure 1:**
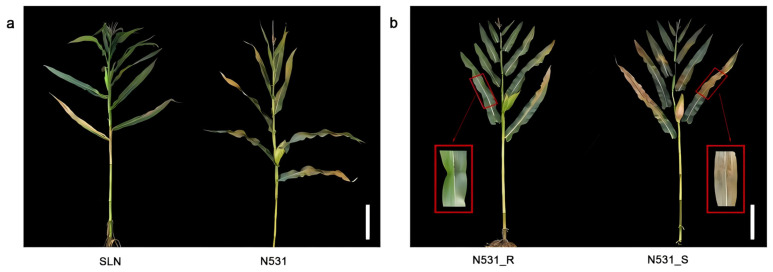
A comparison of resistant and susceptible plants. (**a**) The phenotype of the SLN and N531 maize (*Zea mays* L.) lines; (**b**) the phenotype of resistance to SCR in the resistant line N531_R and susceptible line N531_S were observed. Scale bar: 30 cm.

**Figure 2:**
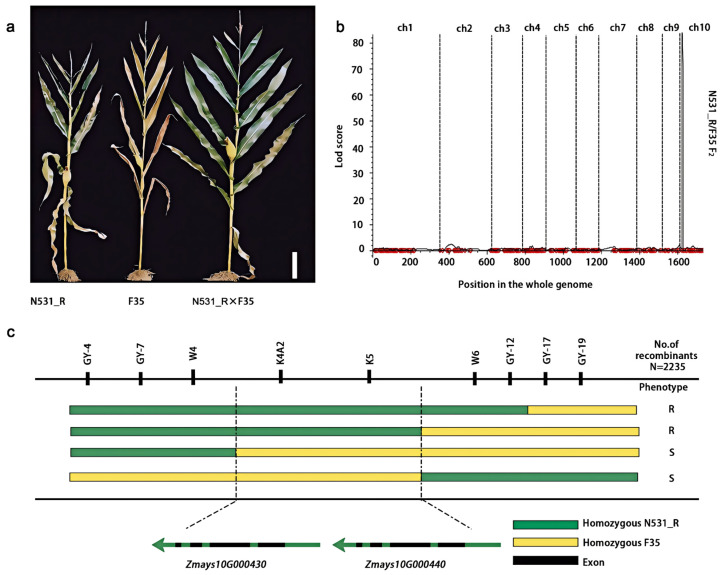
Fine mapping of *RppSLN*. (**a**) The resistant inbred line N531_R and susceptible inbred lines F35 and hybrid F_1_ (N531_R×F35). Scale bar: 30 cm. (**b**) A major southern corn rust-related QTL located in the F_2_ population of N531_R/F35. LOD, logarithm of odds. (**c**) A large-scale isolation of 2253 F_2_ individual plants combined with a genotyping linkage analysis delimited *RppSLN* to a 38 kb interval flanked by the markers W4 and W6. The *RppSLN* region contains two putative NLR genes, which encode NBS-LRR proteins. Green and yellow segments represent N531_R and F35 alleles, respectively. Phenotypes are provided next to the haplotype. R, resistant; S, susceptible.

We identified a landrace, ‘Silunuo’ (SLN), and developed a stable introgression line, SLN-N531 and N531_R, which demonstrated significant resistance to SCR (Figure 1a,b). To verify the genetic contribution of introgression segments derived from SLN, only a preliminary QTL analysis was performed using an F_2_ segregating population of 183 individual plants from the cross of N531_R and the susceptible line F35. Moreover, a major locus was located on the short arm of chromosome 10, with a physical location of approximately 1,285,904–2,260,899 based on the B73v3 reference genome. The LOD score is 84.71 and the phenotypic variation explained (PVE) is 84.77% for resistance to SCR (Figure 2a,b). The numbers of resistant and susceptible plants were 128 and 55, respectively, demonstrating a segregation ratio of 3:1 according to the chi-square test (χ^2^ = 0.89) (Table 1). This suggested that this trait is controlled by a dominant locus or gene. As the resistance locus of N531_R originates from the landrace SLN, we designated it *RppSLN*.

### 3.2. Fine Mapping of RppSLN

To identify the causal gene in *RppSLN*, additional recombinant events were continuously screened. The high-quality reference genomes were assembled in N531_R (Data set1), identifying a substantial number of insertion–deletion (In-Del) markers (Supplementary Materials S1) for further fine mapping. A large-scale F_2_ recombinant event consisting of 2253 individuals was used for further phenotyping and genotyping. Based on this, *RppSLN* was ultimately narrowed down to the interval between the W4 and W6 markers using the two parents N531_R and F35 as resistant and susceptible controls. Based on the N531_R genome sequences, two annotated genes (*Zmays10G000430* and *Zmays10G000440*) are located in a region of approximately 38 kb (Figure 2c), which encodes the NBS-LRR protein.

### 3.3. Identification of Candidate Genes for RppSLN

**Figure 3:**
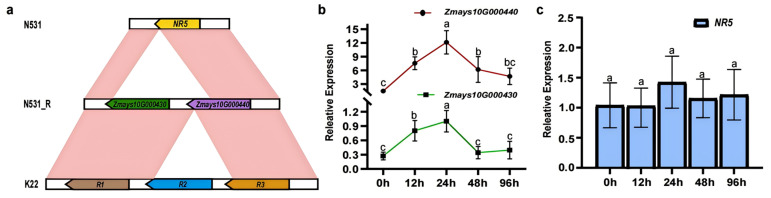
The structure of the candidate genes and their expression levels in *RppSLN*. (**a**) The diagram depicts the structure of *RppSLN* in N531_R, situated between N531 and K22. The top panel reveals that the susceptible line N531 harbors one NBS-LRR gene, noted as *NR5*. The middle panel shows the two candidate genes of *RppSLN* in the resistant line N531_R, annotated as *Zmays10G000430* and *Zmays10G000440*. The bottom panel indicates that the K22 contains three NBS-LRR genes known as *R1*, *R2*, and *R3*. (**b**) An expression analysis of *Zmays10G000430* and *Zmays10G000440* and an expression analysis of *NR5*. The values presented are the means ± standard deviations of the data. For (**b**,**c**), the different letters in the columns indicate a significant difference (*p* < 0.05), as determined by the Duncan test.

We compared the sequences in the candidate region of the susceptible recurrent parent N531 and the resistant introgression line N531_R (Figure 3a). N531 only contains one R gene (*NR5*) (Data set 2), an allele of *Zmays10G000440*. To further confirm the candidate genes, we conducted real-time quantitative polymerase chain reaction (RT-qPCR) analysis detecting the expression levels of these genes (*Zmays10G000430*, *Zmays10G000440*, and *NR5*) between N531_R and its NIL line N531_S at 0 h, 12 h, 24 h, 48 h, and 96 h after infection (Supplementary Materials S1). The results indicated that the relative expression levels of *Zmays10G000430* and *Zmays10G000440* were synergistic expression patterns in N531_R (Figure 3b). However, *NR5* lacks a 2 kb sequence in the 3′-UTR that is shared by *Zmays10G000440*, which might explain why *NR5* lost its resistance. *NR5* expression levels after induction were not significantly changed, suggesting that the absence of the 3′-UTR sequence may impact its function in conferring resistance (Figure 3c). A comparison was made with the published K22 inbred line reference genome [32], which identified three R genes in the target confidence interval (*R1*, *R2*, *R3*). Among these, *R3* was confirmed to be the resistance gene *RppK* for SCR. Further analysis revealed that *Zmays10G000430* and *Zmays10G000440* correspond to the K22 alleles *R1* and *R3*, respectively (Figure 3a).

### 3.4. Transcriptome Expression Pattern Analysis

**Figure 4:**
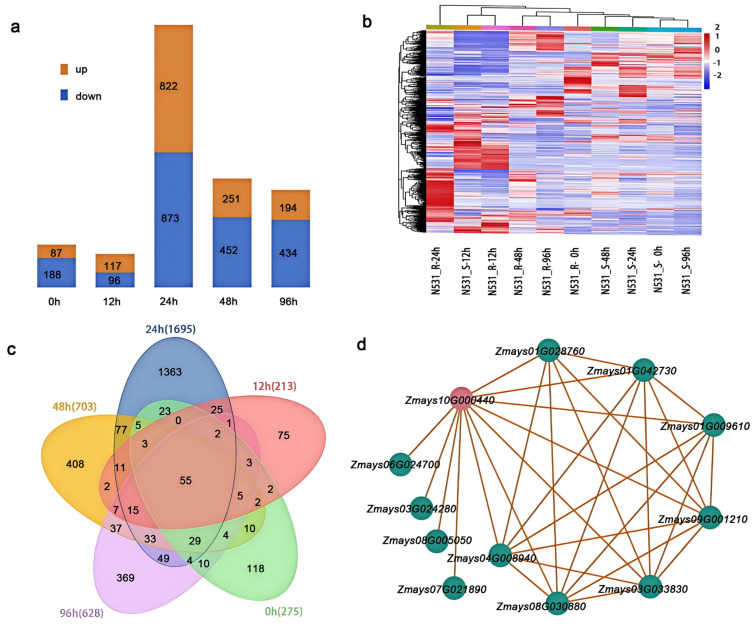
A differential expression gene analysis of N531_R and N531_S. (**a**) The numbers of up- and downregulated DEGs in N531_R compared with N531_S. (**b**) A heatmap of the DEGs among different samples. (**c**) A Venn diagram of DEGs in different treatments. (**d**) A gene co-expression regulatory network analysis of *Zmays10G000440*. The values presented are the means ± standard deviations of the data.

To further elucidate the gene expression profiles in response to pathogen inoculation, we conducted transcriptome sequencing on RNA samples extracted from the leaves of N531_R and N531_S. These samples were collected at multiple time points, immediately before inoculation (0 h) and at 12, 24, 48, and 96 h post-inoculation. A total of 3514 genes were significantly enriched across the five time points, with the majority of expressed genes downregulated in N531_S compared to N531_R (Figure 4a). The expression patterns of the differentially expressed genes (DEGs) in the leaves of N531_R and N531_S across the five sampling stages were visualized using hierarchical clustering (Figure 4b). The expression profiles of the same samples were generally clustered together, such as N531_R samples at 48 and 96 h and N531_S samples at 0, 24, 48, and 96 h showing similar patterns. Notably, the expression profiles of the N531_R samples clustered with the earlier stages of inoculation of the N531_S samples. For instance, the N531_R samples at 12 h clustered with the sample of N531_S at the same point. Further functional categorization of the DEGs was performed according to the Gene Ontology (GO) terms using GOseq tools. The representative categories included defense mechanisms; secondary metabolite biosynthesis, transport, and catabolism; and signal transduction mechanisms. We conducted a hierarchical clustering analysis on differentially expressed gene samples, and the Venn diagram results showed that 15 differentially expressed genes were identified at 12 h, 24 h, 48 h, and 96 h after infection (Figure 4c; Supplementary Materials S2). Additionally, we identified 11 related genes exhibiting similar expression patterns to those of the target candidate genes *Zmays10G000440* (the highest confidence resistance gene) (Figure 4d; Supplementary Materials S3). Among these, *Zmays01G009610* was identified as DNAJ heat shock protein 40 (hsp40). After induction, significant differences in expression levels were observed between N531_R and N531_S at 24 and 48 h post-infection [37,38]. *Zmays01G042730*, which shares similarities with *AtPRL1*, a WD40-containing protein, was significantly induced in N531_S during the first 12 and 24 h following infection [39] (Supplementary Materials S4). This gene may be associated with the enrichment of reactive oxygen species in N531_S.

### 3.5. Physiological and Biochemical Indicators of Resistance and Susceptible Lines in Leaves

**Figure 5:**
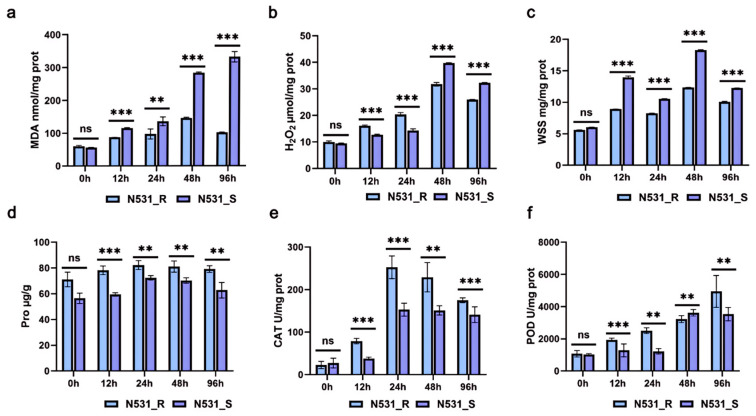
Physiological and biochemical characteristics of N531_R and N531_S at 0 h, 12 h, 24 h, 48 h, and 96 h post-inoculation with SCR. (**a**–**d**) The analysis of leaf samples at 0 h, 12 h, 24 h, 48 h, and 96 h post-inoculation is included in the study, including the measurement of malondialdehyde (MDA), hydrogen peroxide (H_2_O_2_), water-soluble sugar (WSS), and proline content. (**e**,**f**) The analysis of defense enzyme activity, including catalase (CAT) and peroxidase (POD). For (**a**–**f**), the values are presented as the means ± standard deviations of the data. ** *p* < 0.01; *** *p* < 0.001; ns, non-significant (Student’s *t*-test).

Given that genes that respond to stress are closely related to oxidative enzyme activity [40], we measured oxidative stress-related physiological and biochemical indicators in plants. We characterized both NIL N531_R and the susceptible NIL N531_S by investigating changes in the physiological and biochemical parameters of leaves in response to SCR invasion (Supplementary Materials S5). In general, higher levels of MDA (malondialdehyde) (Figure 5a), H_2_O_2_ (hydrogen peroxide) (Figure 5b), and WSS (water-soluble saccharide) (Figure 5c) were found in N531_S seedlings upon SCR infection. Meanwhile, the level of ROS scavenger Pro (proline) in the resistant line N531_R increased more following SCR infection (Figure 5d). The activities of antioxidant enzymes such as CAT (catalase) (Figure 5e) and POD (peroxidase) (Figure 5f) were induced in N531_R seedlings.

### 3.6. Evaluation of Breeding Potential of RppSLN

**Figure 6:**
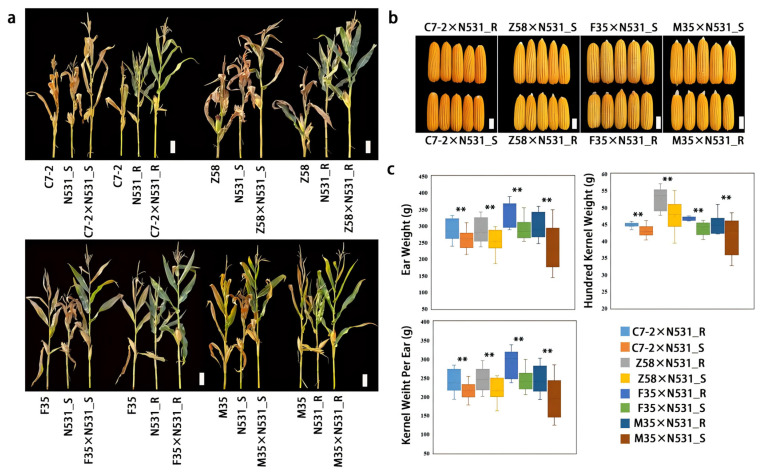
*RppSLN* enhances the yields of maize hybrids under the natural infection of SCR in the field. (**a**) The phenotypes of the hybrids C7-2, Z58, F35, M35 with N531_R, and N531_S under natural infection with SCR in the field. Scale bars: 30 cm. (**b**) The ear phenotypes of the field hybrids C7-2×N531_R, C7-2×N531_S, Z58×N531_R, Z58×N531_S, F35×N531_R, F35×N531_S, M35×N531_R, and M35×N531_S under conditions of SCR. Scale bars: 5 cm. (**c**) The ear weight, single-ear-grain weight, and hundred-grain weight of the hybrids C7-2×N531_R, C7-2×N531_S, Z58×N531_R, Z58×N531_S, F35×N531_R, F35×N531_S, M35×N531_R, and M35×N531_S. The values are presented as the means ± standard deviations of the data. ** *p* < 0.01 (Student’s *t*-test).

To evaluate the breeding potential of *RppSLN* in hybrids, the resistant locus *RppSLN* in N531_R was crossed with the elite parental lines of maize Zhengdan958 (C7-2×Z58) and the susceptible inbred lines F35 and M35 to construct hybrids. Similarly, the susceptible locus within N531_S was used to construct hybrids with the susceptible inbred lines C7-2, Z58, F35, and M35. In the field, the hybrids carrying *RppSLN* showed more resistance to SCR than the susceptible hybrids (Figure 6a,b). We investigated the yield-related traits. The results show that the *RppSLN* gene enhanced maize resistance against SCR and increased ear weight by 9.98%–27.31%, kernel weight per ear by 10.10%-26.25%, and hundred-kernel weight by 4.03%–17.03% (Figure 6c). Overall, *RppSLN* has important production applications for reducing yield losses under SCR conditions and contributed to a yield increase.

## 4. Discussion

### 4.1. Landraces Are Important Germplasm Resources for Maize Improvement

Landraces are crucial sources for developing inbred lines, with the majority of inbred lines derived from these genetically diverse populations and exhibiting superior agronomic traits. The exploration and utilization of these genetic resources enable breeders to develop novel resistant germplasms and cultivars that possess multiple genes that confer resistance to a range of biotic and abiotic stresses, including drought, salinity, and diseases. This approach significantly addresses agricultural disease challenges. For instance, a new rust resistance allelic gene, *Sr22b*, which exhibits significant resistance to the Ug99 race, was discovered in wheat landraces from Romania [41]. Moreover, *YrBDT* has been identified in the Chinese wheat landrace ‘Baidatou’, a new dominant single gene conferring stripe rust resistance [42].

A key strategy for using landrace traits and improving germplasm is to introduce desirable alleles from landraces into breeding materials via backcrossing, creating introgression lines. In our study, we introduced the SCR resistance locus from the landrace SLN into the susceptible inbred line N531, developing the resistant inbred line N531_R. While numerous studies have reported maize inbred lines resistant to SCR, few directly identified resistance genes from landraces for SCR resistance. Our study provided initial evidence demonstrating that the locus conferring resistance to SCR was directly identified from the landrace SLN. However, considering the highly variable physiological races of SCR, this resistance may diminish over time. Thus, identifying additional sources of resistance is essential for maize breeding, which will strengthen disease-resistant breeding efforts and support sustainable agricultural production.

### 4.2. Exploring Resistant Loci That Could Achieve Durable Resistance to SCR by Aggregating Multiple Resistance Genes Is Essential

R-genes enable crops to withstand pathogenic attacks, and they demonstrate considerable variability across different plant species, though they primarily exist in two forms, which are independent loci and clusters [43]. Clustered resistance genes are particularly advantageous for gene sequence exchange, facilitating the emergence of new resistance genes and significantly contributing to expanding resistance gene families [44]. To further verify the existence of a potential synergistic effect between the R genes *Zmays10G000430* and *Zmays10G000440*, we analyzed the time-series expression in the N531_R line. *Zmays10G000430* has a high similarity with *R1* in the *RppK*-resistant locus, which is not the target resistance gene in the K22 inbred line, but along with *Zmays10G000440*, it collectively enhances the resistance expression level in the N531_R line. This finding suggests that multiple resistance genes can synergistically contribute to a more robust defense mechanism against pathogens. Some studies have reported that two NBS-LRR genes mediate rice blast disease in rice. It has also been observed that the deletion or mutation of either of these genes can impact disease resistance. Additionally, *Rpp2A* and *Rpp2B* mediate specialized resistance against downy mildew in Arabidopsis [45], and *Pikm1TS* and *Pikm2TS* collectively mediate resistance against Pikm identified in rice [46], while *RRS1* and *RRS4* mediate resistance against multiple bacterial pathogens [47]. In these instances, either two resistance proteins combine to form a resistance protein complex or one resistance protein acts as an inducing protein or downstream resistance protein for the other. These interactions enhance plant resistance to pathogens and protect plants from disease damage [48].

It is noteworthy that we observed a lower expression level of the only R gene, *NR5*, in the resistant parent after reinfection. However, there is a lack of a shared 3′-UTR between *NR5* and the common amino acids encoded by *Zmays10G000440* and *R3*. As is well known, the untranslated regions (UTRs) of genes play a crucial role in the post-transcriptional regulation of gene expression [49]. This difference could be one of the reasons why *NR5* fails to exhibit the expected resistance. The tandem repeat expression patterns of genes have promising breeding applications, providing a more reliable method for extending the duration of disease resistance in resistance loci. This pattern enhances genes’ potential to maintain robust resistance over time. The pathogen of SCR is highly variable and rapidly generates new physiological microspecies, which can make single R-gene resistance potentially ineffective very quickly. Therefore, a more complex resistance mechanism can be established via the polymerization of multiple R genes, which not only makes it more difficult for the pathogen to break through the resistance but also fights against multiple pathogens through different resistance pathways, and it also effectively enhances durable resistance in maize. In the future, identifying and characterizing additional resistance loci with such gene aggregation patterns may become a key focus in gene identification and breeding strategies, potentially leading to more durable and effective crop disease resistance.

### 4.3. Exploring Network Regulation for Resistance to SCR

Transcriptomic analysis reveals significant differences in gene expression between the resistant and susceptible lines (N531_R and N531_S). This highlights the crucial roles in pathogen defense, the synthesis of defense-related substances, and the regulation of cellular homeostasis [50]. During the early stages of pathogen infection, the balance within plant cells is disrupted. This disruption increases MDA levels, elevates concentrations of ROS such as H_2_O_2_, and heightens defense enzyme activity [51]. In the susceptible line N531_S, the lack of a resistance (R) gene prevents pathogen effector recognition. Consequently, this results in significantly higher levels of ROS and other stress markers compared to the resistant line N531_R. This differential response underscores the importance of the R gene for activating defense mechanisms that mitigate oxidative stress and enhance resistance to pathogen invasion. In this study, we assessed both whether the key candidate gene *Zmays10G000440* confers resistance to SCR and whether a number of key candidate genes exhibit similar expression patterns, finding two genes directly related to the disease resistance effect (*Zmays01G042730* and *Zmays01G009610*).

The gene *Zmays01G042730*, which is similar to *AtPRL1* (a WD40-containing protein), has been identified as a crucial pleiotropic regulator of plant defense responses against both bacterial and fungal pathogens. It plays a significant role in the signaling pathways associated with plant resistance. Research indicates that the knockout or mutation of this gene increases plants’ susceptibility to pathogen infections, highlighting its importance in maintaining plant health [39]. The expression of *Zmays01G042730* is rapidly induced, likely driven by the activity of resistance (R) genes. This rapid response is critical for initiating defense mechanisms. Additionally, the increased activity of catalase (CAT) and peroxidase (POD) oxidases contributes to the maintenance of cellular homeostasis by effectively managing oxidative stress. The significant increase in proline content further mitigates excessive reactive oxygen species accumulation, thus protecting the plant from oxidative damage during pathogen attacks. This coordinated response underscores the essential role of *Zmays01G042730* in enhancing resistance and maintaining cellular integrity under stress conditions. The representative heat shock protein *Zmays01G009610*, classified as HSP40, is a conserved protein known for its robust cellular protective functions. This protein plays a multifaceted role in plant signal transduction, growth, development, and responses to environmental stresses, including heat [37,38]. The overexpression of HSP40 has been shown to enhance antioxidant enzyme activity in the chloroplasts of *Arabidopsis*, thereby reducing damage caused by drought and high-temperature stresses and improving overall stress resistance. Our transcriptomic data align well with physiological and biochemical findings, demonstrating that plants can effectively mitigate potential yield losses during disease outbreaks by expressing specific disease resistance genes. This coordinated response enhances the plant’s ability to activate defense mechanisms, ultimately improving resilience against pathogens. By understanding these relationships, we can better leverage resistance genes in breeding programs to develop crops that maintain productivity even under disease pressure.

Our study identified two candidate genes in the *RppSLN* locus, namely *Zmays10G000430* and *Zmays10G000440*. Future studies will focus on functional assays of these genes to validate their role in SCR resistance. In future research, we will focus on exploring the role of *Zmays10G000430* in downstream functions.

## 5. Conclusions

This study successfully identified a new major SCR resistance locus, *RppSLN*, directly isolated from a landrace, which provides critical insights for expanding disease-resistant germplasm. These candidate genes, along with two R genes, co-expressively were supposed to collectively regulate resistance to SCR in maize.

## Figures and Tables

**Table 1:** Genetic analysis of resistance to SCR.

F_2_ Population	Total	Resistant	Susceptible	Segregation Ratio	χ^2^	*p*-Value
N531_R×F35	183	128	55	2.7	0.89	0.35

## Data Availability

The datasets supporting the results of this article (Data Set1-2) are available at the Chinese Crop Germplasm Resources Information System (CGRIS; https://www.cgris.net/maize/data5/index.html accessed on 10 August 2024).

## Supplementary Materials

The following supporting information can be downloaded at: https://www.mdpi.com/article/10.3390/plants13223227/s1, Supplementary Materials S1. Primers used in this study. Supplementary Materials S2. A total of 15 differentially expressed genes at 12 h, 24 h, 48 h, and 96 h after infection. Supplementary Materials S3. A total of 11 related genes exhibiting similar expression patterns to the target candidate genes *Zmays10G000440*. Supplementary Materials S4. DEGs between N531_R and N531_S. Differential expression analysis of N531_R and N531_S were performed using the R-based DESeq package with a model based on a negative binomial distribution. The resulting *p* values were adjusted using the Benjamini and Hochberg’s approach to control the FDR. Genes with an adjusted *p* < 0.05 were considered differentially expressed. Expression analysis of *Zmays01G009610* (Supplementary Materials S4a) and *Zmays01G042730* (Supplementary Materials S4b). Values are mean SD. ** *p* < 0.05 (Student’s *t*-test). Supplementary Materials S5. Physiological and biochemical characteristics of N531_R and N531_S at 0 h, 12 h, 24 h, 48 h, 96 h after inoculation. Measurement of physiological and biochemical traits of N531_R and N531_S after SCR inoculation. Analysis of leaf samples (L) at 0, 12, 24, 48, and 96 hours post-inoculation. Analysis of defense enzyme activity APX (ascorbate peroxidase) and the contents of O^2−^ (Superoxide anion). Values are mean SD. ** *p* < 0.05 (Student’s *t*-test).
