# Supplementary material for: Identification of RppSLN from an Elite Landrace: A Major Locus Conferring Resistance to Southern Corn Rust in Maize (Zea mays L.)
Source: Plants (Basel). 2024 Nov 16;13(22):3227. doi: 10.3390/plants13223227 (PMC11598374; doi:10.3390/plants13223227)
Supplement: Supplementary file 1 [file plants-13-03227-s001.zip › plants-3261831-supplementary.pdf]

Supplementary Materials S1. Primers used in this study

Marker	Forward primer	Reverse primer
GY-4	CCGCATCCGTCCTCGT	GGAGGTCGACCCCCG
GY-7	CCACCGCATCCGTCCTC	CTCAGCGCGGGGATCTC
W4	AAGCACAGCTCAACATAACTTGAAGGT	CTAGGGTGTTCATCCCAAGAACAATAG
k4A2	CCTTACACAGCTGCTTCGTCATTTC	CGACCTTGTGTTTCTCTTGC
k-5	GGGAGGCTCTGGTGAG	CAGCTGGCTGATGAAGAAAGCAT
W6	AAGGGAAAATTAGTAGAACCACC	TCACCTGTCTGTTTATTACCTCC
GY-12	CCCCCCGACTCCACACC	CAGCAGCCCCCCTCCTC
GY-17	CGCCTAGCTGCTGCTCGTT	GAGATGGCATGGGGTGGAC
GY-19	ATCTTGTGAAGCCATTCCCAT	GAATCAACAAATGCAAGCGAC
N531_R-D-2	CATAAACTCCAAATCGCAGCACT	GCTATTTGGTCTCAGCTTTCCTC
N531-D-8	GGACCTTTCGTGCCCAGAACT	CGACCTGCTCATGGAGGAGTA
Actin	CCCTTCATCACCACGGACTAC	AACCTTCTTGGCACCACCCT

Supplementary Materials S2. A total of 15 differentially expressed genes at 12h, 24h, 48h, and 96h after infection

Gene ID	Annotation
Zmays01G020280	65-kDa microtubule-associated protein
Zmays03G035870	Auxilin-like protein 1
Zmays03G037450	Hypersensitive-induced response protein
Zmays04G007450	Homeobox-leucine zipper protein HOX10
Zmays04G013370	Annexin D5
Zmays06G027420	Anthocyanidin 5,3-O-glucosyltransferase
Zmays06G029600	Type IV inositol polyphosphate 5-phosphatase 3
Zmays07G026380	Replication protein A 70 kDa DNA-binding subunit B
Zmays08G027020	65-kDa microtubule-associated protein 3
Zmays09G000380	Anthranilate O-methyltransferase 2
Zmays09G000430	Metalloendoproteinase 2-MMP
Zmays10G018920	Trafficking protein particle complex II-specific subunit 120 homolog
NewGene_2043	none
NewGene_21454	none
NewGene_2965	none

Supplementary Materials S3. A total of 11 related genes exhibiting similar expression patterns to the target candidate genes Zmays10G000440

Genes ID	Annotation
Zmays01G009610	gene expression, DnaJ homolog subfamily C member 8
Zmays01G028760	mRNA splicing, via spliceosome,N-terminal domain of CBF1 interacting co-repressor CIR
Zmays01G042730	mRNA metabolic process and modification,WD domain, G-beta repeat
Zmays03G024280	mitochondrial electron transport, cytochrome c to oxygen, Energy production and conversion
Zmays03G033830	regulation of embryo sac egg cell differentiation
Zmays04G008940	mRNA processing
Zmays06G024700	mitochondrial electron transport, cytochrome c to oxygen
Zmays07G021890	modification-dependent protein catabolic process
Zmays08G005050	DNA replication
Zmays08G030880	polyglutamine-binding protein 1
Zmays09G001210	gene silencing by RNA-directed DNA methylation

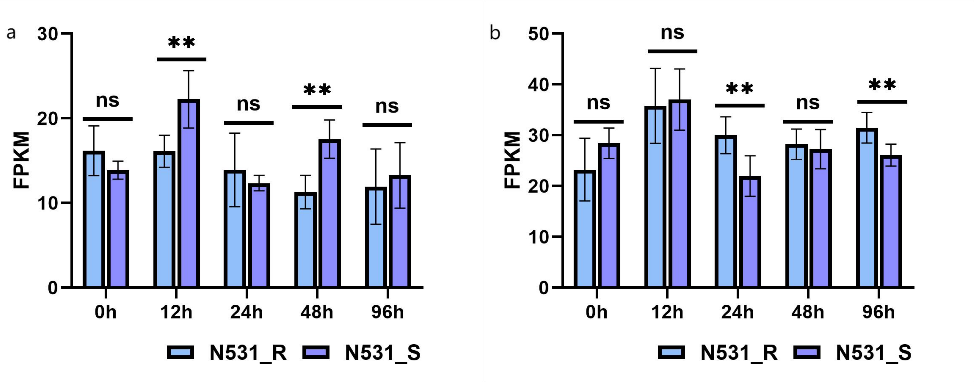

Supplementary Materials S4. DEGs between N531_R and N531_S. Differential expression analysis of N531_R and N531_S were performed using the R-based DESeq package with a model based on a negative binomial distribution. The resulting P values were adjusted using the Benjamini and Hochberg's approach to control the FDR. Genes with an adjusted P<0.05 were considered differentially expressed. Expression analysis of *Zmays01G009610* (Supplementary Materials S4-a) and *Zmays01G042730* (Supplementary Materials S4-b). Values are mean SD. **P < 0.05 (Student's *t*-test)

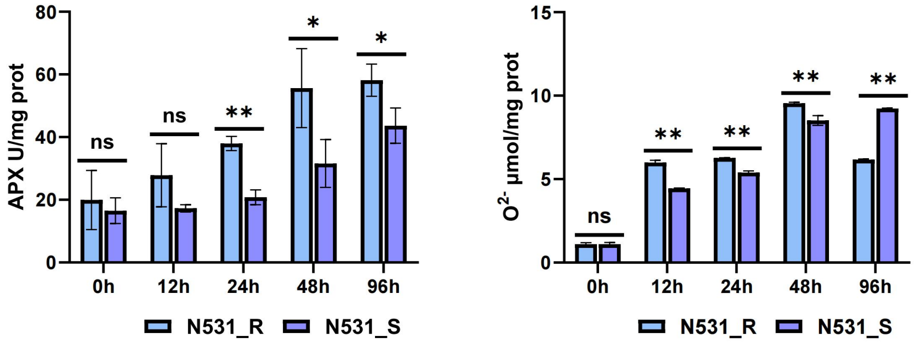

Supplementary Materials S5. Physiological and biochemical characteristics of N531_R and N531_S at 0h, 12h, 24h, 48h, 96h after inoculation.Measurement of physiological and biochemical traits of N531_R and N531_S after SCR inoculation. Analysis of leaf samples (L) at 0, 12, 24, 48, and 96 hours post-inoculation. Analysis of defense enzyme activity APX (ascorbate peroxidase) and the contents of O^{2-} (Superoxide anion). Values are mean SD. **P < 0.05 (Student's *t*-test).
